# Validation of Energy and Nutrition Intake in Japanese Elderly Individuals Estimated Based on a Short Food Frequency Questionnaire Compared against a 7-day Dietary Record: The Kyoto-Kameoka Study

**DOI:** 10.3390/nu11030688

**Published:** 2019-03-22

**Authors:** Daiki Watanabe, Hinako Nanri, Tsukasa Yoshida, Miwa Yamaguchi, Mayu Sugita, Yoshizu Nozawa, Yuki Okabe, Aya Itoi, Chiho Goto, Yosuke Yamada, Kazuko Ishikawa-Takata, Hisamine Kobayashi, Misaka Kimura, Kyoto-Kameoka Study Group

**Affiliations:** 1 Section of Healthy Longevity Research, National Institutes of Biomedical Innovation, Health and Nutrition, Tokyo 162-8636, Japan; watanabe.smusm.1021@gmail.com (D.W.); t-yoshida@nibiohn.go.jp (T.Y.); yamaguchi3005@gmail.com (M.Y.); yamaday@nibiohn.go.jp (Y.Y.); 2 Department of Pharmacology, St. Marianna University School of Medicine, Kanagawa 216-8511, Japan; 3 Department of Health and Sports Sciences, Kyoto Gakuen University, Kyoto 621-8555, Japan; misaka@kyotogakuen.ac.jp; 4 Laboratory of Applied Health Sciences, Kyoto Prefectural University of Medicine, Kyoto 602-8566, Japan; 5 Senior Citizen’s Welfare Section, Kameoka City Government, Kyoto 621-8501, Japan; 6 Ajinomoto Co., Inc., Tokyo 210-8681, Japan; mayu_sugita@ajinomoto.com (M.S.); yoshizu_nozawa@ajinomoto.com (Y.N.); yuki_okabe@ajinomoto.com (Y.O.); hisamine_kobayashi@ajinomoto.com (H.K.); 7 Department of Health, Sports and Nutrition, Faculty of Health and Welfare, Kobe Women’s University, Hyogo 650-0046, Japan; aitoi@kwjc.kobe-wu.ac.jp; 8 Department of Health and Nutrition, Faculty of Health and Human Life, Nagoya Bunri University, Aichi 492-8520, Japan; goto.chiho@nagoya-bunri.ac.jp; 9 Department of Nutrition and Metabolism, National Institutes of Biomedical Innovation, Health and Nutrition, Tokyo 162-8636, Japan; kazu@nibiohn.go.jp

**Keywords:** food frequency questionnaire, dietary record, age, sex, measurement error

## Abstract

To determine the association between geriatric disorders and dietary intake, validation of a food frequency questionnaire (FFQ) for elderly individuals is needed. We compared energy and nutrient intakes derived from dietary records (DR) and FFQ in an elderly population and compared the data against results from middle-aged individuals (30–68 years) from a previous study. Current participants included 65 women and 78 men (65–88 years) who completed FFQ and 7-day DR in a subpopulation of the Kyoto-Kameoka study. Our FFQ was created for middle-aged individuals. To validate the FFQ, we investigated equivalent precision by comparing the correlation coefficients between the present and previous study. Median correlations for energy and nutrient intake between the DR and FFQ in the current and previous studies were 0.24 and 0.30 (*p* = 0.329) in women and 0.24 and 0.28 (*p* = 0.399) in men, respectively. The median ratio of FFQ to DR for these intakes were also similar. The accuracy and precision of the FFQ for energy and nutrient intake in elderly individuals did not differ compared with previous findings in a middle-aged population. A validation study evaluating energy and nutrient intake using recovery biomarkers is further needed.

## 1. Introduction

In population-based epidemiologic studies, dietary intake is commonly assessed using self-administered dietary assessment methods such as dietary records (DRs) [1], food frequency questionnaires (FFQs) [2], and face-to-face 24-hour dietary recalls (24HRs) [3]. In particular, FFQs have been widely used in large-scale population-based studies owing to easy administration, less burden on participants and staff, and low cost compared to other assessment methods [4]. FFQs consist of a list of food items with response categories to indicate the usual frequency of consumption over a certain time, and estimated total energy and nutrient intakes are calculated by frequency of consumption of each food item, with consideration of portion size [5]. FFQs are suitable for evaluating individual food and nutrient consumption and for ranking individuals according to the distribution of intake [6]. Systematic reviews have reported that the energy and nutrients intake estimated by FFQ were moderate correlations those estimated using DRs and 24HRs [7]. However, estimated measurements from FFQs-derived data suffer from random and systematic errors (i.e., subject age, sex, and portion size) and may not adequately represent habitual food intake because of wide variations in dietary habits within different populations in general middle-aged people [7]. Dietary survey-associated measurement error factors have been reported for age [8,9], sex [9,10], body mass index (BMI) [11,12,13], and socioeconomic status [14]. Additionally, a previous study showed that dietary intake was associated with factors regarding family status [15], appetite [16], and dysphagia [17]. During assessment of dietary intake by FFQs these variables have been found to be associated with misreporting of the dietary intake by the subject and declining physical function, which may affect the results. Therefore, it is necessary to clarify whether these variables can contribute to dietary measurement error in FFQs.

To assess habitual Japanese diets, Tokudome et al. developed a widely used, comprehensive, self-administered, short FFQ for the general Japanese population using 46 food and beverage items [18]. A previous study reported validation of this FFQ against a 3-day DR (*n* = 202) [19,20,21] and plasma fatty acid (*n* = 177) [22] in community-dwelling middle-aged (range: 30–68 years) individuals. Dietary sodium, potassium, and saturated fatty acid intake estimated using this FFQ are related to all-cause mortality and cardiovascular death [23,24]. In addition, in the Kyoto-Kameoka study using this FFQ, we reported that frequency of consumption of some foods (i.e., fruit and vegetable or protein-rich foods [seafood and dairy products]) is associated with geriatric disorders such as oral health-related quality of life [25] and frailty [26]. Although we reported an association between the frequency of consumption of specific foods and geriatric disorders, the precision of ranking elderly individuals according to dietary intake using this FFQ has not been validated [21]. Of note, a general FFQ may underestimate energy and nutrition intake compared with estimates derived from DRs or biomarkers [27]. Moreover, a recent systematic review suggested that the validity of general FFQs should be reevaluated [28]. Therefore, for each new population group assessed, there is a need for a validation study using an FFQ [29]. 

Using results from a self-administered FFQ and 7-day DR in Japanese elderly individuals, we primarily aimed to evaluate mean intake and rank individuals with regard to energy and nutrition intake compared with that of the previous study by Tokudome et al. [21]. The secondary objective was to investigate the association between variables including age and dietary measurement errors with energy and nutrient intake based on the DR and FFQ.

## 2. Materials and Methods

### 2.1. Study Design and Participants

The Kyoto-Kameoka Longitudinal Study is based on a community-based prospective cohort, which included 13,294 individuals aged ≥65 years living in Kameoka City, Kyoto Prefecture, Japan on 29 July 2011 [25,26,30,31,32,33]. *The Needs in the Sphere of Daily Life survey* (baseline survey) was initially conducted in this cohort by postal mail. Secondly, *the Health and Nutrition Status Survey* (additional survey), which includes assessments such as food intake (assessed by FFQ), nutritional status, oral health, and activities of daily living, was conducted on 14 February 2012 (during winter in Japan). This survey included 11,985 individuals, of whom some were excluded owing to certification of long-term care (levels 1, 2) or support (levels 1, 2) (*n* = 1240) or because they died (*n* = 69) between 29 July 2011 and 13 February 2012. Among the included participants, 8319 participants submitted valid responses to this additional survey. In 8319 participants, the 1379 participants who completed the physical check-up examination in the Kyoto-Kameoka study were informed about the design of the present study and were invited to participate in May 2012 [31]. In total, 175 participants volunteered and provided informed consent. Of these participants, 147 individuals conducted a 7-consecutive day DR. One hundred and forty-three participants completed the 7-day DR in May and June 2012. Those who did not complete it were excluded (*n* = 3). Finally, a total of 65 women and 78 men aged 65–85 years and 66–88 years, respectively, completed both the FFQ and 7-day DR. These participants were included in the present analysis.

This study protocol was approved by the Ethics Committee of Kyoto Prefectural University of Medicine (RBMR-E-363) and the National Institute of Health and Nutrition (NIHN187-3).

### 2.2. Dietary Assessment

#### 2.2.1. Food Frequency Questionnaire

The FFQ was administered using the validated questionnaires of Tokudome et al. [19,20,21], and this questionnaire was collected by postal mail in *the Health and Nutrition Status Survey* (additional survey). To assess mean dietary intake in the group, the FFQ asked participants the frequency of consumption of 46 food and beverage items over the past year. However, semi-quantitative portion sizing was not considered, and the FFQ did not provide detailed information on portion sizes for each food. Portion sizes were uniformly determined according to age (middle-aged adults) and sex. Food and beverage consumption frequencies were classified into eight categories: (1) never or seldom, (2) 1–3 times per month, (3) 1–2 times per week, (4) 3–4 times per week, (5) 5–6 times per week, (6) once per day, (7) twice per day, and (8) 3 or more times per day. The weights assigned to each intake frequency category were 0, 0.1, 0.2, 0.5, 0.8, 1.0, 2.0, and 3.0, respectively. The energy and nutrient intake was calculated using a program developed at the Department of Public Health, Nagoya City University School of Medicine, based on *the Standard Tables of Food Composition in Japan* (fifth revised edition) [34].

#### 2.2.2. Dietary Records

We collected DR over 7 consecutive days during May and June 2012 (during spring in Japan) to include both weekdays and weekends. During the informational meeting, research staff (registered dietitians) were educated regarding how to administer the DR to participants, using completed dietary record sheets as examples. Each participant was provided blank record sheets to record their DR, a digital scale (TANITA, Tokyo, Japan), and paper media for education. The research dietitians instructed the participants to record every item of food and beverage consumed daily at or between meals. If the food or beverage items were difficult to measure (e.g., prepared at a restaurant or bought at a supermarket or convenience store), we instructed participants to record the size and quantity of foods as precisely as possible, using household measures.

The dietitians checked all completed records at the participant’s home and reviewed them at least twice in a standardized manner. The completed DRs were coded and entered by the research dietitians into the energy and nutrient analysis program entitled WELLNESS21 software (TopBusinessSystem, Okayama, Japan), which conforms to *the Standard Tables of Food Composition in Japan* [35]. If the recorded foods and beverages by the participants were not listed in *the Standard Tables of Food Composition in Japan*, these were replaced by similar foods.

### 2.3. Statistical Analysis

Energy and nutrient intakes are higher in men than women. The difference in the accuracy and precision of estimating energy and nutrient intake by sex, necessitates sex-stratified evaluation. This was employed in the current study while evaluating the mean dietary intake in this cohort and ranking individuals according to dietary intake. Previous validation studies have also employed the sex-stratified model [21], with all statistical analyses performed separately for women and men. Data distribution and normality (skewness and kurtosis) were checked before analysis. Variables such as “change in body weight in the past 3 months”, “socioeconomic status”, “family status”, “appetite”, and “swallowing function” were extracted from *the Needs in the Sphere of Daily Life survey* (baseline survey) and *the Health and Nutrition Status survey* (additional survey), the details of which have been described elsewhere [25,26,30,31,32,33]. Body weight was measured by the research staff during the physical check-up examination in the Kyoto-Kameoka study, using a standard weight scale (OMRON, Tokyo, Japan) to the nearest 0.1 kg, with participants wearing light clothing and no shoes. BMI was calculated as body weight (kg) divided by the square of self-reported body height (m^2^). Continuous variables and categorical variables are reported as mean ± standard deviation (SD) and as number (%), respectively, to show the characteristics of variables. Identified missing answers or logical errors were handled as missing data. Correlation analyses were performed using the *phi* coefficient and Spearman’s correlation coefficient for one nominal and one continuous variable and for two continuous variables, respectively. To evaluate the correlation coefficients of energy and nutrient intake, energy and 23 selected nutrients were calculated in accordance with a previously reported validation study [21], and the median correlation coefficient for these intakes were used [36]. Our FFQ was developed based on individual foods which contribute to 85% of within-person variance for energy and nutrient intake in the middle-aged population. It is unclear whether the FFQ could evaluate intakes in the elderly similar to that of middle-aged people since it was not originally designed for the elderly. To confirm the validation of the FFQ in an elderly population, we compared the results with that of community-dwelling middle-aged adults (range: 30–68 years) from a previous study [21]. To validate the FFQ, equivalent precision using an equation developed by Meng et al. was used to compare correlation coefficients from the present and previous study [37]. This equation is used to compare between two correlation coefficients, and we applied it for the purpose of evaluating the difference in precision of ranking individuals according to energy and nutrients intake between elderly and middle-aged adults using this FFQ. According to the Medical Care System in Japan, elderly age groups are classified as those between 65–74 and ≥75 years; this accounts for differences in insurance premiums for medical treatment [38]. Moreover, some epidemiologic studies have used a cutoff age of 75 years for misreporting of energy intake [39] or estimation of nutrients intake [40]. Therefore, we also examined the association between dietary measurement errors due to FFQ and age-stratified (<75 and ≥75 years) models using the Mann-Whitney U test. These variables are reported as the median (interquartile range [IQR]). Dietary measurement error due to the FFQ was calculated as follows: (energy or nutrients intake estimated from FFQ/energy or nutrients intake estimated from 7-day DR). 

The between-person variance (σ_b_^2^) and within-person variance (σ_w_^2^) for intake were calculated using one-way analysis of variance. The group size (G_S_) needed to estimate the “true” mean intake with a 95% confidence interval (CI) within the specified percent of deviation (D_0_) required for DR was calculated using the following equation [41,42]: G_S_ = 1.96^2^ × [(CV_b_^2^ + CV_w_^2^)/D_0_^2^], where CV_b_^2^ and CV_w_^2^ are the between-person variance and within-person variance, respectively, and D_0_ is used as the specified percent of deviation as 2.5%, 5%, 10%, or 20%. The number of days (ND_1_) needed to estimate a specified correlation coefficient (*r*) between observed and unobserved individual’s usual (“true”) mean intakes required for DR was calculated using the following equation [41,42]: ND_1_ = [*r*^2^/(1 − *r*^2^)] × VR, where VR is the variance ratio as determined by σ_w_^2^/σ_b_^2^. Based on this analysis, *r* is thus a reliability indicator regarding the classification or ranking of individuals in a population, and it used specified correlation coefficients of 0.75, 0.80, 0.85, 0.90, and 0.95. The number of days (ND_2_) needed to estimate an individual’s "true" mean intake with a 95% CI within the specified percent of deviation required for DR was calculated using the following equation [41,42]: ND_2_ = (1.96 × σ_w_^2^/D_1_)^2^, where D_1_ is the specified percent of deviation of 5%, 10%, 20, or 30%. A *p* value <0.05 for two-sided tests was considered statistically significant. All analyses were conducted using the JMP Pro software package (SAS institute Inc., Cary, NC, USA) for Windows.

## 3. Results

Characteristics of the study participants are shown in Table 1. Mean age and mean BMI of women and men were 72.5 (range: 65 to 85) and 73.8 (range: 66 to 88) years and 22.6 (range: 16.8 to 31.1) and 22.9 (range: 14.3 to 30.0) kg/m^2^, respectively. A change of 3 kg or less body weight in the past three months occurred in 65 (100%) women and 74 (96.1%) men. Co-habiting was a characteristic of 53 (81.6%) women and 74 (94.9%) men. 

The comparison of mean intakes and the correlation coefficients on energy intake and intake of 23 selected nutrients assessed using the DR and FFQ in the current and previous studies are shown in Table 2 and Table 3. The median correlations of energy and nutrient intake between the current and previous studies were 0.24 and 0.30 (*p* = 0.329) and 0.24 and 0.28 (*p* = 0.399) in women and men, respectively. However, compared with the previous study, the current study exhibited a significantly lower correlation coefficient for fat, energy-dense carbohydrates, and n-3 polyunsaturated fatty acids intake in men, and energy-dense fat, carbohydrate, and iron intake in women. Compared with the value estimated using the DR in the current study, energy intake estimated using the FFQ was underestimated in both sexes (mean energy intake ± SD from the DR and FFQ, respectively were: 1790 ± 220 and 1562 ± 343 kcal/day and 2070 ± 301 and 1863 ± 528 kcal/day for women and men, respectively. However, the difference was not statistically significant *p* >0.05. In addition, the current study underestimated energy-providing nutrients and some other nutrients. These findings are similar to the results of the previous study, and tended to underestimate the same nutrients. In addition, the median ratio of FFQ to DR for energy and nutrient intake were not different between the current and previous study. These ratios were 0.88 and 0.86 and 0.90 and 0.79 among women and men in the current and previous study, respectively. 

The comparison between the energy and nutrient intake estimated of the FFQ and the DR in men according to age (<75 and ≥75 years) in the current study is shown in Supplemental Table S1. Mean energy intake estimated using the DR was higher than that estimated using the FFQ in those aged <75 years (2151 ± 288 and 1783 ± 568 kcal/day for the DR and FFQ, respectively) but not in those aged ≥75 years (1946 ± 282 and 1985 ± 443 kcal/day for the DR and FFQ, respectively). In addition, those aged ≥75 years exhibited a significantly higher ratio for energy intake between the FFQ to DR compared with those aged <75 years (ratio [IQR] of FFQ to DR for energy intake: 0.88 [0.72 to 1.01] and 1.05 [0.95 to 1.15] for those aged <75 and ≥75 years, *p* < 0.001). Moreover, there were similar differences in the ratio of FFQ to DR for energy-providing nutrients and some nutrients such as cholesterol, iron, and soluble dietary fiber. However, there were no differences in ratio of FFQ to DR for energy and 23 selected nutrients between the age groups (<75 and ≥75 years) in women (Supplemental Table S2).

Supplemental Tables S3–S6 presents the number of days and group size required to ensure accuracy for the group and individual usual (“true”) mean intake by DR according to sex group. The larger within-person or between-person variance ratio implies that many DR days would be required to rank individuals according to dietary intake. In the present study, older women and men would need to be evaluated for 2 to 8 and 2 to 7 days, respectively, to achieve a correlation coefficient (*r*) of within 0.90 between the observed and usual (“true”) mean intake based on DR (Supplemental Table S4). The larger within-person variance means that many DR days would be required to evaluate an individual’s usual dietary intake. For instance, older women and men would need 7 to 594 and 10 to 465 days, respectively, to achieve within 10% deviation (Supplemental Table S3).

Table 4 presents the Spearman’s correlation coefficients for the measurement error of energy and nutrient intakes and other variables in both women and men. In men, there was a moderate correlation between the measurement error of energy and energy-providing nutrients and age (Spearman rank correlation coefficients: 0.41, 0.34, 0.28, and 0.38; *p* < 0.01 for energy, protein, fat, and carbohydrate intake, respectively). In addition, measurement error of some nutrients such as cholesterol, iron, and soluble dietary fiber were also associated with age. Conversely, no significant correlation was observed for energy and nutrient intakes and age in women.

## 4. Discussion

In this study, we assessed the validity of the FFQ used for the Kyoto-Kameoka Longitudinal Cohort Study among the elderly Japanese population and compared the results with those of a previously validated study conducted by Tokudome et al. among the middle-aged Japanese population [21]. In regard to the correlation between the DR and FFQ, median correlation coefficients for energy and nutrient intake between the current and previous studies were not significantly different. In addition, the median ratio of FFQ to DR for energy and nutrient intake were not different between the current and previous study. Moreover, the measurement error tended to increase in older men; however, this association was not observed in women.

FFQs tend to have a larger measurement error for underestimating energy and nutrient intake compared with the error associated with DR or 24-hour dietary recalls, using biomarkers included doubly labeled water and urine collection as a reference [43]. Validation studies are therefore important because the true intake value is not accurately determined by FFQs [29]. However, in the present analysis, the median correlation coefficients for energy and nutrient intake between the DR and FFQ were not significantly different between middle-aged and elderly populations in either gender (Table 2 and Table 3). Compared with the previous study, the current study demonstrated a significantly lower correlation for fat, energy-dense carbohydrates, and n-3 polyunsaturated fatty acids intake in men and energy-dense fat and carbohydrate and iron intake in women (Table 2 and Table 3). Each food item included in FFQs is an aggregate of different foods with different nutrient contents. To accurately reflect the diet and foods actually eaten in the target population, these nutrient contents use the weighted mean value of all foods included in the item [44]. Accordingly, the food list, portion size, and weighted mean nutrient contents may be important factors for precision of ranking and accuracy of assessing intra-group energy and nutrient intake using FFQs. In the present study, the ability to rank energy and nutrient intake did not differ between elderly and middle-aged individuals (Table 2 and Table 3). We speculate that habitual food and beverage consumption patterns between elderly and middle-aged people were probably not different. In the previous Japanese study, self-administered dietary assessment questionnaires were developed for the middle-aged population and were validated by evaluating individual food and nutrient consumption and by ranking individuals according to the distribution of intake in elderly people (aged 80 years or older) [45]. It may therefore be presumed that Japanese middle-aged adults have already established their dietary habits. Consequently, evaluation of individual food and nutrient consumption and individual ranks were not different between elderly and middle-aged people. In comparison, studies evaluating within-person and between-person variance for energy and nutrient intake in Japanese younger and older adults have demonstrated that older adults have smaller variances than younger adults [41]. For these reasons, it may be presumed that older individuals may be more established in their dietary habits than younger subjects [7,46]. The larger within-person or between-person variance ratio means that many DR days would be required to evaluate individual ranks according to dietary intake. In the present study, older women and men would need 2 to 8 and 2 to 7 days, respectively, to achieve a correlation coefficient (*r*) of within 0.90 between observed and usual (“true”) mean intake based on DR (Supplemental Table S4). A previous study has reported that the repeatability of nutrient intakes estimated using FFQs were higher in older adults [7,46]. This study provided a sufficient number of days as a reference value for comparing the precision of FFQ and DR; the individual ranks according to dietary intake could be evaluated with a correlation coefficient (*r*) of within almost 0.90 between observed and usual (“true”) mean intakes.

The ratio of FFQ to DR for energy and nutrient intake were not different between the current and previous study (Table 2 and Table 3). A recent systematic review reported that the validity of FFQs should be re-evaluated because energy, carbohydrate, calcium, and vitamin C intakes were overestimated more often in women than in men [28]. Although energy and nutrient intake estimated using the FFQ was similarly underestimated in both sexes, compared with the value estimated using the DR in the current study, these findings are similar to the results of the previous study. Our FFQ was developed based on foods that contribute to 85% of within-person variance for each energy and nutrient intake in the middle-aged population [18]. In addition, the FFQ used in the current study listed less food items compared with that of other FFQs (median 79 items, range 5 to 350) mentioned in a systematic review [29]. Short dietary survey questionnaires tend to underestimate energy and nutrient intake compared with that of longer questionnaires and DR [47]. Therefore, this FFQ considered DR as reference and tended to underestimate energy and nutrient intake owing to the development method. The larger within-person variance implies that many DR days would be required to evaluate an individual’s usual dietary intake. For instance, older women and men would need 7 to 594 and 10 to 465 days, respectively, to achieve within 10% deviation (Supplemental Table S3). Canadian adults have demonstrated larger within-person variances with respect to other nutrients than energy intake [48]. Estimates of intake of nutrients rather than energy may provide a markedly inadequate estimate of the usual intake among individuals. Therefore, our results do not provide accurate and precise estimates for some nutrients (i.e., vitamin A, vitamin D, and polyunsaturated fatty acids). More accurate estimation of these nutrients will require comparison to biomarkers as reference. Estimated nutrients intake with larger within-person variances necessitates the use of an alternative method including a validated FFQ that can estimate the usual dietary intakes of the group over a longer period than DR or 24HR. This should take into consideration the study design, target-population characteristics, and staff or participant burden [41].

In the present study, dietary measurement errors were moderately correlated with age in men (Table 4). It therefore appears that older age is associated with measurement errors. Some studies have shown that measurement errors in dietary assessment is associated with age [8,9]; this may be due to differences in portion size. Gazan et al. [49] speculated that systematic errors in FFQ-estimated food and nutrient intake may be a result of inaccuracy in portion sizes used or nutrient content in food items from the relevant composition tables. Pfrimer et al. [9] reported that elderly populations use smaller food portion sizes, including those for rice, meat, and milk, compared with the portion sizes of middle-aged populations. Compared with reference methods of dietary assessment such as DR and 24HR, smaller portion sizes in the elderly may overestimate dietary intake, which is estimated using uniform portion sizes irrespective of age in the FFQ. The positive correlation between dietary measurement errors and age may be explained by the smaller portion size of the food and beverage items in the questionnaire that are taken by the elderly. Energy and nutrient intake may be influenced not only by portion size but also by foods and beverages listed in FFQs. In the present study, male participants aged ≥75 years had a significantly lower number of food items consumed per day compared with those aged <75 years (24.9 in those aged <75 years and 21.8 in those aged ≥75 years, *p* = 0.031), but this was not true in women (24.6 in those aged <75 years and 22.5 in those aged ≥75 years, *p* = 0.133). If the middle-aged population consumes more food items than does the elderly population, an intra-group comparison of FFQ-derived habitual dietary intake will be inaccurate owing to the lower number of listed food items [50]. It is therefore necessary to determine whether new food lists, portion size, and weighted mean nutrient contents for elderly populations improve ranking ability and estimation of mean intake of energy and nutrients compared to food lists and weighted mean nutrient contents created for middle-aged populations.

Our study has several limitations. First, there was an interval of 2–3 months between the FFQ and the DR. There can be seasonal variations in dietary and food intake, but these seasonal variations are greatest between summer and winter [51,52,53]. Therefore, although there may be a seasonal difference in the results of the FFQ and the DR, it is not likely to be substantial. Second, some previously reported dietary measurement error factors (physical activity [12,14], education [14], race/ethnicity [14], and motivation [54]) were not evaluated in our study. In addition, gender-based differences in these factors were unclear in this cohort. However, there are no differences in race or ethnicity among the Japanese population. Third, it is unknown whether elderly individuals have the same ability to remember and quantify the frequency of food consumption as middle-aged participants, because the FFQs cannot evaluate the cognitive and memory functions. However, the 7-day DR was independently conducted among our study participants. Our FFQ used even portion sizes based on those from the middle-aged adult group according to sex, because there was no method to semi-quantitatively determine portion size for each participant. Thus, if the study participants could correctly conduct complex work such as completing the DR, we considered that they should also be able to record the frequency of consumption of each food and beverage category. A previous study comprising 36 men and 44 women validated the assessment of dietary intake in a Japanese population aged 80 years or older. The study used a self-administered dietary questionnaire that asked participants only regarding the frequency of consumption [45]. It may therefore be valid to assess dietary intake using an FFQ irrespective of declining cognitive and memory functions. Fourth, our study administered a 7-day DR. In contrast, the study by Tokudome et al. [21] administered a 3-day DR (two weekdays and one weekend day). The difference in the number of days may influence the results. To eliminate the weighted difference between weekdays and the weekend, the current study included five weekdays and two weekend days, thus any differences were not likely to be substantial. However, this may affect the results when evaluating individual food and nutrient consumption and for ranking individuals according to the distribution of intake (Supplemental Table S4). Moreover, estimated energy and nutrient intake may differ between the FFQ and DR if they are estimated using a different edition of the standard table of food consumption in Japan. Although this standard table includes a larger amount of food and beverage items with an analysis of energy and nutrient values, the listed food and beverage items remain largely unchanged from the previous edition. Fifth, the present study had a small sample size; therefore, it may not reflect the mean energy intake of the population. However, we were able to evaluate the accuracy and precision of energy and nutrient intake and confirmed that the sample size was almost sufficient to assess mean intake of the group (Supplemental Tables S4–S6). However, further study is needed for some nutrients (i.e., vitamin A, vitamin D, and polyunsaturated fatty acids). In addition, the present study may harbor selection bias, as participants were not randomly chosen and may have exhibited a higher level of health consciousness. These limitations may hamper the generalizability of the results. In addition, we excluded three participants who did not complete a 7-day DR. Mennen et al. has assumed that the dietary recall of participants who completed a dietary survey is more precise (smaller intra-individual variation) than that of participants who dropped out [55]. Thus, generalizability may be limited owing to overestimation of correlation coefficients. Nevertheless, the present results show a larger, age-dependent difference between DR and FFQs in men. The inclusion of health-conscious people in this study may have contributed to underestimation of the relationship between age and measurement errors of the FFQ. Moreover, in this self-reported dietary assessment, we were unable to evaluate and compare objective biomarkers for dietary assessment with DR and FFQ. A previous study reported that total energy expenditure as measured using the doubly labeled water method; energy intake estimated from DR and FFQ were underestimated by approximately 20 and 30%, respectively [27]. It is possible that the DR we used as a reference may have been an underestimation. Consequently, the present results might exhibit a higher degree of underestimation compared with the true mean energy intake. Tinker et al has reported that FFQ based-estimated energy intake by developed calibration equation based on multiple regression analysis, which included variables affecting self-reporting biases using biomarker is associated with the incidence of diabetes but not when uncalibrated [56]. It is therefore important to evaluate the accuracy and precision of energy and nutrient intake using objective recovery biomarkers such as doubly labeled water, urinary samples, and serum concentrations.

## 5. Conclusions

Our results suggest that the accuracy and precision of the FFQ for energy and nutrient intake in elderly individuals did not differ compared with findings from a previous study in a middle-aged population. To further evaluate energy and nutrient intake, development of a calibration equation to adjust for self-reported bias using biomarkers is needed. This approach could be expected to further our understanding of the relation between dietary intake and disease risk, while substantially addressing measurement error problems that have long plagued the field of nutritional epidemiology.

## Figures and Tables

**Table 1:** Characteristics of study participants according to sex.

	Women (*n* = 65)			Men (*n* = 78)			Total (*n* = 143)		
Age (years) ^a^	72.5	± 4.8		73.8	± 5.6		73.2	± 5.3	
≥75 years ^b^	22	(33.8)		31	(39.7)		53	(37.0)	
Body height (cm)	150	± 5.1		164	± 5.4		158	± 5.3	
Body weight (kg)	51.1	± 7.6		61.9	± 8.8		57.0	± 8.3	
BMI (kg/m^2^) ^a,c^	22.6	± 3.5		22.9	± 2.9		22.8	± 3.2	
<18.5 ^b^	7	(10.8)		6	(7.7)		13	(9.1)	
18.6–24.9	42	(64.6)		49	(62.8)		91	(63.6)	
≥25.0	16	(24.6)		23	(29.5)		39	(27.3)	
Change in body weight in past 3 month ^b,d^									
No change	46	(70.8)		53	(67.9)		99	(69.2)	
Socioeconomic status ^b,e^									
High	34	(52.3)		41	(52.5)		75	(52.4)	
Low	29	(44.6)		36	(46.2)		65	(45.5)	
Family status ^b,f^									
Single	11	(16.9)		4	(5.1)		15	(10.5)	
Co-habiting	53	(81.6)		74	(94.9)		127	(88.8)	
Appetite ^b,g^									
Good	62	(95.4)		76	(97.4)		138	(96.5)	
Poor	1	(1.5)		2	(2.6)		3	(2.1)	
Swallowing function ^b,e^									
Good	6	(9.2)		4	(5.1)		10	(7.0)	
Poor	57	(87.7)		73	(93.6)		130	(90.9)	

Abbreviations: BMI, body mass index. ^a^ Continuous values are shown as mean ± standard deviation. ^b^ Categorical values are shown as number (percentage). ^c^ BMI was calculated as body weight in kilograms divided by the square of height in meters (kg/m^2^). ^d^ Missing; women (*n* = 1) and men (*n* = 1). ^e^ Missing; women (*n* = 2) and men (*n* = 1). ^f^ Missing; women (*n* = 1). ^g^ Missing; women (*n* = 2).

**Table 2:** Comparison of daily intakes of energy and nutrients measured using dietary records compared with those of a short food frequency questionnaire in women.

Nutrients	Unit	Current Study (C) (*n* = 65)						Tokudome et al. Study [21] (T) (*n* = 129)						Ratio		Spearman’s		*p* Value ^d^
		7-day DR			FFQ			3-day DR			FFQ			C ^a^	T ^b^	C *r* ^c^	T *r* ^c^	
		Mean		SD	Mean		SD	Mean		SD	Mean		SD					
Energy	(kcal)	1790	±	220	1562	±	343	1924	±	332	1639	±	186	0.87	0.85	0.40	0.37	0.422
Protein	(g)	69.3	±	10.8	52.8	±	13.2	74.5	±	16.3	55.2	±	7.8	0.76	0.74	0.29	0.30	0.463
Fat	(g)	52.2	±	11.7	52.9	±	17.0	59.2	±	16.5	48.4	±	9.6	1.01	0.82	0.08	0.22	0.183
Carbohydrate	(g)	256	±	35	216	±	58	265	±	50	227	±	36	0.85	0.86	0.38	0.45	0.285
Protein ^e^	(%)	15.5	±	1.7	13.7	±	2.3	15.5	±	2.0	13.5	±	1.5	0.88	0.87	0.29	0.37	0.285
Fat ^e^	(%)	26.2	±	4.5	31.0	±	9.0	27.5	±	5.1	26.7	±	4.9	1.18	0.97	0.04	0.33	**0.024**
Carbohydrate ^e^	(%)	57.2	±	4.7	54.8	±	6.7	55.2	±	6.1	55.2	±	5.0	0.96	1.00	0.23	0.45	**0.050**
SFA	(g)	13.2	±	3.6	12.2	±	2.9	16.0	±	5.5	12.4	±	2.5	0.93	0.78	0.11	0.35	0.053
MUFA	(g)	16.5	±	4.4	19.9	±	6.8	19.8	±	6.2	16.9	±	3.4	1.21	0.85	0.05	0.12	0.319
PUFA	(g)	11.9	±	2.7	16.6	±	6.4	14.0	±	4.1	13.5	±	2.9	1.40	0.97	0.08	0.05	0.420
n-6 PUFA	(g)	9.6	±	2.3	14.7	±	5.9	11.0	±	3.4	11.5	±	2.6	1.53	1.04	0.01	0.20	0.106
n-3 PUFA	(g)	2.2	±	0.7	2.4	±	0.9	2.8	±	1.1	2.2	±	0.5	1.09	0.80	0.19	0.17	0.442
MO n-3 PUFA ^f^	(g)	0.8	±	0.5	0.5	±	0.2	0.9	±	0.7	0.7	±	0.2	0.61	0.78	0.31	0.29	0.457
Cholesterol	(mg)	308	±	89	246	±	109	345	±	132	264	±	64	0.80	0.76	0.17	0.15	0.439
Iron	(mg)	8.2	±	1.9	7.0	±	2.2	8.9	±	2.7	7.7	±	1.6	0.86	0.86	0.25	0.50	**0.029**
Calcium	(mg)	541	±	162	556	±	168	609	±	231	566	±	144	1.03	0.93	0.38	0.33	0.350
Carotene	(µg)	4157	±	2679	2316	±	662	4241	±	2103	3550	±	1131	0.56	0.84	0.23	0.31	0.288
Vitamin A ^g^	(µg RE)	602	±	395	942	±	611	1067	±	832	1052	±	422	1.57	0.99	0.16	0.22	0.340
Vitamin D	(µg)	9.7	±	4.7	4.4	±	1.6	8.0	±	5.9	7.2	±	2.6	0.45	0.91	0.24	0.25	0.463
α-tocopherol	(mg)	7.8	±	2.0	10.5	±	3.6	9.4	±	3.0	8.6	±	1.8	1.36	0.92	0.31	0.00	**0.019**
Vitamin B1	(mg)	0.92	±	0.22	0.68	±	0.11	1.04	±	0.30	0.70	±	0.10	0.74	0.65	0.15	0.13	0.451
Vitamin B2	(mg)	1.15	±	0.25	1.18	±	0.34	1.38	±	0.43	1.20	±	0.20	1.03	0.89	0.40	0.38	0.426
Folate	(µg)	345	±	96	353	±	116	409	±	164	384	±	93	1.02	0.94	0.31	0.29	0.448
Vitamin C	(mg)	137	±	59	115	±	44	136	±	69	122	±	34	0.84	0.90	0.36	0.43	0.288
SDF	(g)	3.0	±	0.7	2.0	±	0.6	2.4	±	0.7	2.3	±	0.5	0.66	0.96	0.13	0.28	0.161
IDF	(g)	10.8	±	2.5	7.9	±	2.1	12.0	±	3.7	9.0	±	1.9	0.74	0.75	0.32	0.32	0.491
TDF	(g)	14.7	±	3.3	10.7	±	2.9	16.6	±	5.1	12.4	±	2.7	0.73	0.75	0.28	0.34	0.330
Median														0.88	0.86	0.24	0.30	0.329

Abbreviations: DR, dietary record; FFQ, food frequency questionnaire; IDF, insoluble dietary fiber; MO n-3 PUFA, marine origin n-3 PUFA; MUFA, monounsaturated fatty acids; PUFA, polyunsaturated fatty acids; SD, standard deviation; SDF, soluble dietary fiber; SFA, saturated fatty acids; TDF, total dietary fiber. ^a^ Ratio of FFQ to 7-day DR [Current study] (C). The age range was 65 to 85 years. ^b^ Result of ratio or correlation coefficient between FFQ and 3-day DR in a previous study by Tokudome et al. (T) [21]. The age range of women was 30 to 68 years. ^c^ Spearman rank correlation analysis for crude values. ^d^ Comparison of correlation coefficients between the Tokudome study [21] and current studies (calculated as described by Meng et al. [37]). Bold values are statistically significant (*p* < 0.05). ^e^ Value are shown as energy-dense macronutrient (% energy intake). ^f^ Sum of eicosapentaenoic acid (20:5), docosapentaenoic acid (22:5), and docosahexaenoic acid (22:6). ^g^ Sum of retinol, β-carotene/12, α-carotene/24, and cryptoxanthin/24.

**Table 3:** Comparison of daily intakes of energy and nutrients measured using dietary records compared to that of a short food frequency questionnaire in men.

Nutrients	Unit	Current Study (C) (*n* = 78)						Tokudome et al. Study [21] (T) (*n* = 73)						Ratio		Spearman’s		*p* Value ^d^
		7-day DR			FFQ			3-day DR			FFQ			C ^a^	T ^b^	C *r* ^c^	T *r* ^c^	
		Mean		SD	Mean		SD	Mean		SD	Mean		SD					
Energy	(kcal)	2070	±	301	1863	±	528	2342	±	469	1987	±	268	0.90	0.85	0.19	0.36	0.138
Protein	(g)	77.2	±	13.1	57.0	±	16.0	88.4	±	22.1	60.8	±	10.2	0.74	0.69	0.19	0.22	0.430
Fat	(g)	56.3	±	14.9	50.9	±	16.9	66.1	±	22.6	47.1	±	11.9	0.90	0.71	-0.01	0.38	**0.007**
Carbohydrate	(g)	286	±	45	265	±	100	313	±	58	293	±	52	0.93	0.94	0.43	0.57	0.125
Protein ^e^	(%)	14.9	±	1.6	12.6	±	2.7	15.1	±	2.0	12.3	±	1.4	0.84	0.81	0.20	0.38	0.120
Fat ^e^	(%)	24.4	±	4.9	26.5	±	11.2	25.1	±	5.4	21.4	±	4.6	1.09	0.85	0.15	0.49	**0.011**
Carbohydrate ^e^	(%)	55.4	±	5.9	54.6	±	10.9	53.9	±	6.2	58.8	±	4.6	0.99	1.09	0.48	0.68	**0.031**
SFA	(g)	14.1	±	4.5	11.9	±	3.4	16.6	±	6.6	11.3	±	2.0	0.84	0.68	0.24	0.35	0.235
MUFA	(g)	17.8	±	5.1	19.1	±	7.1	23.1	±	9.3	17.5	±	4.4	1.07	0.76	-0.01	0.12	0.214
PUFA	(g)	13.2	±	4.0	16.7	±	5.8	16.4	±	5.3	14.1	±	3.2	1.27	0.86	-0.01	0.05	0.366
n-6 PUFA	(g)	10.8	±	3.6	14.8	±	5.6	12.8	±	4.5	11.8	±	2.7	1.37	0.92	-0.02	0.20	0.090
n-3 PUFA	(g)	2.4	±	0.7	2.4	±	0.9	3.3	±	1.2	2.3	±	0.5	1.01	0.70	0.11	0.37	**0.048**
MO n-3 PUFA ^f^	(g)	0.9	±	0.5	0.5	±	0.2	1.1	±	0.9	0.7	±	0.3	0.60	0.65	0.06	0.28	0.085
Cholesterol	(mg)	347	±	114	215	±	52	424	±	176	274	±	64	0.62	0.65	0.35	0.15	0.104
Iron	(mg)	8.5	±	2.0	6.8	±	1.7	9.8	±	2.4	7.7	±	1.9	0.80	0.79	0.32	0.38	0.348
Calcium	(mg)	526	±	182	539	±	184	592	±	186	508	±	129	1.02	0.86	0.49	0.21	**0.024**
Carotene	(µg)	3632	±	2661	2630	±	822	4244	±	1840	3229	±	1285	0.72	0.76	0.27	0.18	0.283
Vitamin A ^g^	(µg RE)	567	±	536	992	±	644	989	±	478	1052	±	384	1.75	1.06	0.09	0.10	0.475
Vitamin D	(µg)	8.7	±	4.7	4.8	±	2.0	9.4	±	5.4	7.4	±	3.4	0.55	0.79	0.24	0.33	0.288
α-tocopherol	(mg)	7.9	±	2.3	10.5	±	3.0	10.1	±	3.3	8.6	±	2.1	1.33	0.85	0.15	0.16	0.484
Vitamin B1	(mg)	0.97	±	0.22	0.66	±	0.14	1.18	±	0.40	0.69	±	0.08	0.68	0.58	0.05	0.19	0.191
Vitamin B2	(mg)	1.18	±	0.30	1.07	±	0.34	1.48	±	0.44	1.12	±	0.21	0.90	0.76	0.48	0.34	0.162
Folate	(µg)	337	±	115	319	±	84	417	±	148	357	±	109	0.95	0.86	0.27	0.21	0.355
Vitamin C	(mg)	122	±	71	97	±	33	123	±	57	103	±	34	0.80	0.84	0.31	0.24	0.315
SDF	(g)	3.1	±	1.1	2.2	±	0.7	3.7	±	1.2	2.1	±	0.6	0.69	0.57	0.39	0.28	0.227
IDF	(g)	10.8	±	3.2	8.5	±	2.5	12.1	±	3.2	8.0	±	2.2	0.78	0.66	0.29	0.22	0.315
TDF	(g)	15.0	±	4.2	11.5	±	3.4	16.6	±	4.4	11.4	±	3.1	0.77	0.69	0.33	0.34	0.486
Median														0.90	0.79	0.24	0.28	0.399

Abbreviations: DR, dietary record; FFQ, food frequency questionnaire; IDF, insoluble dietary fiber; MO n-3 PUFA, marine origin n-3 PUFA; MUFA, monounsaturated fatty acids; PUFA, polyunsaturated fatty acids; SD, standard deviation; SDF, soluble dietary fiber; SFA, saturated fatty acids; TDF, total dietary fiber. ^a^ Ratio of FFQ to 7-day DR [Current study] (C). The age range was 66 to 88 years. ^b^ Result of ratio or correlation coefficient between FFQ and 3-day DR in a previous study by Tokudome et al. (T) [21]. The age range of women was 30 to 68 years. ^c^ Spearman rank correlation analysis for crude values. ^d^ Comparison of correlation coefficients between the Tokudome study [21] and current studies (calculated as described by Meng et al. [37]). Bold values are statistically significant (*p* < 0.05). ^e^ Value are shown as energy-dense macronutrient (% energy intake). ^f^ Sum of eicosapentaenoic acid (20:5), docosapentaenoic acid (22:5), and docosahexaenoic acid (22:6). ^g^ Sum of retinol, β-carotene/12, α-carotene/24, and cryptoxanthin/24.

**Table 4:** Spearman rank correlation analysis between the measurement error and variables according to sex ^a^.

Nutrients	Women (*n* = 65)							Men (*n* = 78)						
	Age	BMI	Weight Change	Economic	Family Status	Appetite	Dysphagia	Age	BMI	Weight Change	Economic	Family Status	Appetite	Dysphagia
	*r* ^b^	*r* ^b^	*r* ^b^	*r* ^b^	*φ* ^c^	*φ* ^c^	*φ* ^c^	*r* ^b^	*r* ^b^	*r* ^b^	*r* ^b^	*φ* ^c^	*φ* ^c^	*φ* ^c^
Energy	0.01	0.00	0.19	−0.15	−0.10	−0.02	0.03	0.41 ***	−0.04	0.04	0.21	−0.10	−0.12	0.14
Protein	0.12	0.12	0.11	−0.14	−0.15	0.04	0.09	0.34 **	−0.02	−0.04	0.07	−0.05	−0.10	0.16
Fat	0.04	0.03	0.04	0.00	−0.09	−0.16	0.07	0.28 **	−0.04	−0.11	0.00	0.10	0.02	0.14
Carbohydrate	−0.11	0.01	0.19	−0.23	−0.13	0.05	−0.01	0.38 **	−0.02	0.08	0.20	−0.07	−0.15	0.13
Protein ^d^	0.08	0.18	−0.11	0.06	−0.10	0.09	0.08	−0.03	−0.03	−0.04	−0.19	0.16	0.03	0.00
Fat ^d^	0.02	−0.04	−0.10	0.10	−0.05	−0.12	0.03	0.00	−0.01	−0.11	−0.07	0.23*	0.11	0.01
Carbohydrate ^d^	−0.16	0.03	0.10	−0.23	−0.09	0.10	−0.04	0.07	0.07	0.03	0.10	−0.04	−0.12	0.07
SFA	0.21	0.01	0.01	0.00	0.00	−0.07	0.04	0.27 *	0.04	−0.11	0.05	0.01	0.11	0.05
MUFA	−0.02	−0.03	−0.06	0.01	−0.10	−0.16	0.01	0.33 **	−0.02	−0.08	0.01	0.11	0.05	0.10
PUFA	−0.03	−0.06	0.07	−0.08	−0.10	−0.16	0.15	0.31 **	−0.02	−0.09	0.01	0.06	−0.03	0.16
n−6 PUFA	0.01	−0.06	0.06	−0.09	−0.07	−0.12	0.13	0.33 **	0.00	−0.09	0.04	0.03	−0.02	0.14
n−3 PUFA	−0.04	−0.02	0.07	0.02	−0.13	−0.25 *	0.12	0.14	−0.06	0.05	−0.17	0.04	−0.12	0.12
MO n−3 PUFA	0.02	−0.09	0.14	0.16	−0.11	−0.08	0.11	−0.09	−0.01	0.17	−0.27	−0.07	−0.13	−0.03
Cholesterol	0.04	−0.04	0.04	−0.10	−0.07	−0.07	0.08	0.23 *	0.10	0.13	−0.04	0.12	0.01	0.02
Iron	0.06	0.07	0.21	−0.06	−0.30 *	0.05	0.18	0.43 ***	−0.01	−0.04	0.01	0.02	−0.19	0.06
Calcium	0.18	0.09	0.20	0.04	−0.09	0.02	0.04	0.09	−0.01	0.04	0.08	−0.10	0.00	0.10
Carotene	0.02	0.19	0.36 **	−0.03	−0.28 *	0.04	−0.14	0.18	0.16	−0.09	0.01	0.12	−0.16	−0.09
Vitamin A	0.09	0.09	0.17	−0.02	−0.20	0.05	0.12	0.00	0.10	−0.04	−0.01	0.04	0.00	−0.05
Vitamin D	−0.06	0.28 *	0.06	0.07	−0.25 *	0.09	0.03	0.08	−0.14	0.18	−0.19	−0.15	−0.11	0.10
α-tocopherol	0.06	0.11	0.13	0.00	−0.19	−0.05	0.04	0.31 **	0.06	−0.11	−0.07	0.15	−0.17	0.06
Vitamin B1	0.11	0.37 **	0.19	0.16	−0.24	0.02	−0.01	0.28 *	−0.07	−0.20	−0.15	0.03	0.00	0.10
Vitamin B2	0.19	0.18	0.29 *	0.08	−0.14	0.04	0.07	0.22	0.02	−0.03	−0.02	0.02	−0.20	0.07
Folate	0.20	0.18	0.27 *	0.14	−0.15	−0.07	0.09	0.23 *	0.04	0.03	−0.06	−0.01	−0.10	0.04
Vitamin C	0.08	0.03	0.28 *	0.11	−0.15	−0.12	0.01	0.30 **	0.15	0.12	0.05	0.05	−0.04	0.10
SDF	0.08	0.14	0.23	−0.06	−0.20	−0.01	0.03	0.29 *	−0.07	−0.11	0.07	−0.01	−0.16	0.19
IDF	0.04	0.13	0.19	0.02	−0.21	0.01	−0.14	0.13	−0.07	0.07	−0.03	−0.06	−0.15	0.13
TDF	0.01	0.15	0.24	−0.04	−0.21	−0.01	0.00	0.16	−0.12	0.05	0.02	0.00	−0.15	0.20

Abbreviations: DR, dietary record; FFQ, food frequency questionnaire; IDF, insoluble dietary fiber; MO n-3 PUFA, marine origin n-3 PUFA; MUFA, monounsaturated fatty acids; PUFA, polyunsaturated fatty acids; SDF, soluble dietary fiber; SFA, saturated fatty acids; TDF, total dietary fiber. ^a^ Dietary measurement error due to FFQ was calculated as follows: (energy or nutrient intake estimated from FFQ / energy or nutrients intake estimated from 7-day DR). To assess the measurement error of energy and nutrients intake by FFQ, correlation analyses were performed between dietary measurement error (equation described above) and variables (age [continuous data], BMI [continuous data], weight change [categorical data], socioeconomic status [categorical data], family status [categorical data], appetite [categorical data], and dysphagia [categorical data]). The level of statistical significance is indicated with a single asterisk (*) if *p* < 0.05, two (**) if *p* < 0.01, and three (***) if *p* < 0.001. If the results presented a positive correlation, these variables are overestimated factors related to energy and nutrient intake (conversely, a negative correlation indicates that they underestimate it). ^b^ Spearman’s correlation coefficient. ^c^ Phi coefficient (φ). ^d^ Values are shown as energy-dense macronutrient (% energy intake).

## Supplementary Materials

The following are available online at https://www.mdpi.com/2072-6643/11/3/688/s1. Table S1: Comparison of daily energy and nutrient intake based on dietary records or a short food frequency questionnaire according to age-stratified groups in men, Table S2: Comparison of daily energy and nutrient intake based on dietary records or a short food frequency questionnaire according to age-stratified groups in women, Table S3: The coefficients of variation, and within- to between-person variance ratios of energy and nutrient intake based on dietary record (DR), shown per sex, Table S4: Group size required to ensure accuracy of energy and nutrients intake with 95% confidence interval (CI) within the specified % deviation (D_0_) of a group’s mean from the group’s usual (“true”) mean intake based on dietary record (DR), shown per sex, Table S5: Number of days required to ensure precision of energy and nutrients intake within specified correlation coefficient (*r*) between observed and usual (“true”) mean intake based on dietary record (DR), shown per sex, Table S6: Number of days required to ensure accuracy of energy and nutrients intake with 95% confidence interval (CI) within the specified % deviation (D_1_) of an individual’s usual (“true”) mean intake based on dietary record (DR), shown per sex.
