# Supplementary material for: Validation of Energy and Nutrition Intake in Japanese Elderly Individuals Estimated Based on a Short Food Frequency Questionnaire Compared against a 7-day Dietary Record: The Kyoto-Kameoka Study
Source: Nutrients. 2019 Mar 22;11(3):688. doi: 10.3390/nu11030688 (PMC6471352; doi:10.3390/nu11030688)
Supplement: Supplementary file 1 [file nutrients-11-00688-s001.pdf]

Table S1. Comparison of daily energy and nutrient intake based on dietary records or a short food frequency questionnaire according to age-stratified groups in men.

		<75 years (*n* = 47)								≥75 years (*n* = 31)								
		7-day DR			FFQ			Difference [a]		7-day DR			FFQ			Difference [a]		*p* Value [b]
Nutrients	Unit	Mean		SD	Mean		SD	Ratio		Mean		SD	Mean		SD	Ratio		
Energy	(kcal)	2151	±	288	1783	±	568	0.88	(0.72 to 1.01)	1946	±	282	1985	±	443	1.05	(0.95 to 1.15)	**<0.001**
Protein	(g)	80.6	±	12.1	55.2	±	15.7	0.72	(0.57 to 0.83)	72.0	±	12.9	59.8	±	16.3	0.84	(0.72 to 0.97)	**0.004**
Fat	(g)	60.6	±	14.8	51.0	±	15.7	0.84	(0.64 to 1.00)	49.9	±	12.8	50.6	±	18.9	0.96	(0.76 to 1.29)	**0.041**
Carbohydrate	(g)	291	±	46	246	±	108	0.92	(0.63 to 1.05)	277	±	43	294	±	81	1.07	(0.95 to 1.19)	**<0.001**
Protein [c]	(%)	15.1	±	1.6	12.9	±	3.1	0.83	(0.72 to 0.96)	14.8	±	1.6	12.0	±	1.8	0.82	(0.73 to 0.92)	0.646
Fat [c]	(%)	25.3	±	4.9	28.5	±	12.2	1.04	(0.76 to 1.46)	23.0	±	4.7	23.5	±	9.0	0.94	(0.77 to 1.17)	0.574
Carbohydrate [c]	(%)	54.2	±	5.5	52.1	±	11.9	1.02	(0.88 to 1.12)	57.2	±	6.2	58.4	±	7.8	1.04	(0.93 to 1.12)	0.432
SFA	(g)	15.1	±	4.6	12.1	±	3.6	0.83	(0.66 to 0.93)	12.6	±	3.8	11.7	±	3.0	0.96	(0.69 to 1.22)	**0.026**
MUFA	(g)	19.2	±	5.2	18.8	±	6.6	0.91	(0.75 to 1.24)	15.7	±	4.0	19.6	±	7.8	1.07	(0.87 to 1.66)	**0.039**
PUFA	(g)	14.2	±	4.1	16.3	±	5.4	1.14	(0.86 to 1.52)	11.7	±	3.3	17.4	±	6.4	1.40	(1.14 to 2.08)	**0.020**
n-6 PUFA	(g)	11.7	±	3.7	14.3	±	5.1	1.25	(0.88 to 1.67)	9.4	±	2.9	15.5	±	6.3	1.52	(1.16 to 2.23)	**0.012**
n-3 PUFA	(g)	2.5	±	0.7	2.3	±	0.7	0.95	(0.71 to 1.30)	2.2	±	0.7	2.5	±	1.1	1.13	(0.76 to 1.33)	0.181
MO n-3 PUFA [d]	(g)	0.9	±	0.5	0.5	±	0.2	0.67	(0.40 to 1.01)	0.8	±	0.5	0.5	±	0.2	0.59	(0.43 to 1.48)	0.554
Cholesterol	(mg)	366	±	115	214	±	55	0.59	(0.46 to 0.75)	319	±	110	216	±	49	0.70	(0.57 to 0.79)	**0.038**
Iron	(mg)	8.9	±	2.2	6.4	±	1.8	0.76	(0.62 to 0.89)	7.9	±	1.6	7.3	±	1.4	0.95	(0.74 to 1.10)	**<0.001**
Calcium	(mg)	554	±	172	562	±	201	1.01	(0.87 to 1.21)	484	±	193	504	±	149	1.10	(0.85 to 1.42)	0.275
Carotene	(μg)	3975	±	2986	2624	±	867	0.78	(0.51 to 1.11)	3112	±	2009	2640	±	761	0.89	(0.64 to 1.56)	0.153
Vitamin A [e]	(μg RE)	643	±	666	1078	±	702	1.82	(1.26 to 2.83)	451	±	188	862	±	531	1.51	(1.16 to 2.89)	0.752
Vitamin D	(μg)	9.2	±	4.3	4.6	±	1.6	0.53	(0.36 to 0.70)	7.9	±	5.4	5.0	±	2.6	0.77	(0.38 to 1.42)	0.098
α-tocopherol	(mg)	8.5	±	2.4	10.2	±	2.7	1.25	(0.96 to 1.51)	7.0	±	1.9	10.8	±	3.5	1.47	(1.27 to 1.99)	**0.006**
Vitamin B1	(mg)	1.02	±	0.21	0.65	±	0.14	0.63	(0.58 to 0.70)	0.90	±	0.23	0.67	±	0.14	0.74	(0.56 to 0.97)	**0.022**
Vitamin B2	(mg)	1.25	±	0.29	1.07	±	0.38	0.84	(0.69 to 0.99)	1.08	±	0.29	1.06	±	0.27	0.95	(0.83 to 1.22)	**0.014**
Folate	(μg)	358	±	120	313	±	82	0.89	(0.77 to 1.13)	304	±	101	328	±	87	0.92	(0.81 to 1.47)	**0.045**
Vitamin C	(mg)	130	±	75	91	±	30	0.84	(0.54 to 0.97)	109	±	62	107	±	36	1.03	(0.70 to 1.55)	**0.008**
SDF	(g)	3.3	±	1.1	2.1	±	0.7	0.64	(0.46 to 0.83)	2.9	±	0.9	2.2	±	0.6	0.79	(0.62 to 0.98)	**0.010**
IDF	(g)	11.3	±	3.5	8.5	±	2.6	0.73	(0.60 to 0.97)	10.1	±	2.4	8.3	±	2.2	0.78	(0.65 to 1.01)	0.397
TDF	(g)	15.6	±	4.7	11.4	±	3.7	0.71	(0.56 to 0.95)	14.1	±	3.2	11.6	±	2.9	0.81	(0.68 to 0.92)	0.159
Median								0.84	(0.72 to 1.01)							0.95	(0.81 to 1.07)	0.064

Abbreviations: DR, dietary record; FFQ, food frequency questionnaire; IDF, insoluble dietary fiber; MO n-3 PUFA, marine origin n-3 PUFA; MUFA, monounsaturated fatty acids; PUFA, polyunsaturated fatty acids; SD, standard deviation; SDF, soluble dietary fiber; SFA, saturated fatty acids; TDF, total dietary fiber. [a] Difference = (FFQ / 7-day DR). The ratios are shown median (interquartile range). [b] The Mann-Whitney U test was used to compare measurement error

between participants aged <75 and ≥75 years. Bold values are statistically significant ($p < 0.05$). [c] Value are shown as energy-dense macronutrient (% energy intake). [d] Sum of eicosapentaenoic acid (20:5), docosapentaenoic acid (22:5), and docosahexaenoic acid (22:6). [e] Sum of retinol, β-carotene/12, α-carotene/24, and cryptoxanthin/24.

Table S2. Comparison of daily energy and nutrient intake based on dietary records or a short food frequency questionnaire according to age-stratified groups in women.

		<75 years (*n* = 43)								≥75 years (*n* = 22)								
		7-day DR			FFQ			Difference [a]		7-day DR			FFQ			Difference [a]		*p* Value [b]
Nutrients	Unit	Mean		SD	Mean		SD	Ratio		Mean		SD	Mean		SD	Ratio		
Energy	(kcal)	1779	±	210	1543	±	398	0.87	(0.77 to 0.99)	1813	±	243	1601	±	199	0.86	(0.80 to 0.99)	0.613
Protein	(g)	69.1	±	11.1	52.0	±	14.1	0.74	(0.61 to 0.87)	69.7	±	10.5	54.5	±	11.5	0.81	(0.68 to 0.91)	0.335
Fat	(g)	52.6	±	12.5	52.0	±	17.2	1.05	(0.69 to 1.29)	51.3	±	10.0	54.6	±	16.8	1.05	(0.86 to 1.30)	0.425
Carbohydrate	(g)	253	±	35	213	±	67	0.86	(0.71 to 0.99)	261	±	36	222	±	32	0.84	(0.78 to 0.91)	0.961
Protein [c]	(%)	15.6	±	1.9	13.7	±	2.4	0.86	(0.78 to 0.97)	15.4	±	1.1	13.6	±	2.3	0.91	(0.74 to 1.01)	0.884
Fat [c]	(%)	26.6	±	4.8	31.1	±	9.2	1.13	(0.87 to 1.48)	25.5	±	3.6	30.7	±	8.7	1.15	(0.99 to 1.47)	0.672
Carbohydrate [c]	(%)	56.9	±	5.2	54.4	±	7.2	0.97	(0.89 to 1.06)	57.7	±	3.6	55.5	±	5.4	0.95	(0.89 to 1.02)	0.528
SFA	(g)	13.2	±	3.7	11.9	±	2.6	0.95	(0.71 to 1.18)	13.1	±	3.5	12.9	±	3.3	1.06	(0.86 to 1.25)	0.190
MUFA	(g)	16.6	±	4.6	19.9	±	7.0	1.22	(0.84 to 1.56)	16.2	±	4.2	20.0	±	6.7	1.19	(1.00 to 1.54)	0.787
PUFA	(g)	12.0	±	2.7	16.6	±	6.6	1.34	(1.07 to 1.71)	11.6	±	2.6	16.8	±	5.9	1.37	(1.02 to 2.13)	0.672
n-6 PUFA	(g)	9.7	±	2.4	14.6	±	6.1	1.53	(1.10 to 1.85)	9.3	±	2.3	15.0	±	5.5	1.52	(1.22 to 2.42)	0.425
n-3 PUFA	(g)	2.2	±	0.7	2.4	±	0.9	1.06	(0.87 to 1.42)	2.2	±	0.6	2.4	±	0.9	1.00	(0.83 to 1.45)	0.613
MO n-3 PUFA [d]	(g)	0.8	±	0.5	0.5	±	0.2	0.78	(0.48 to 1.06)	0.8	±	0.4	0.5	±	0.2	0.59	(0.42 to 0.96)	0.328
Cholesterol	(mg)	311	±	85	240	±	100	0.68	(0.56 to 0.91)	303	±	98	258	±	127	0.77	(0.53 to 1.18)	0.537
Iron	(mg)	8.2	±	2.1	7.1	±	2.4	0.82	(0.59 to 1.08)	8.1	±	1.5	7.0	±	1.8	0.87	(0.72 to 1.03)	0.632
Calcium	(mg)	553	±	164	556	±	183	1.03	(0.79 to 1.21)	519	±	159	556	±	139	1.01	(0.92 to 1.34)	0.442
Carotene	(µg)	4159	±	3029	2311	±	628	0.64	(0.44 to 0.96)	4153	±	1877	2327	±	740	0.58	(0.43 to 0.82)	0.467
Vitamin A [e]	(µg RE)	622	±	462	839	±	351	1.44	(1.09 to 2.08)	562	±	211	1145	±	909	1.44	(1.07 to 3.26)	0.613
Vitamin D	(µg)	9.8	±	5.2	4.4	±	1.5	0.46	(0.35 to 0.71)	9.4	±	3.5	4.2	±	1.8	0.43	(0.34 to 0.60)	0.575
α-tocopherol	(mg)	7.8	±	2.2	10.4	±	3.8	1.27	(1.00 to 1.61)	7.6	±	1.6	10.8	±	3.3	1.34	(1.10 to 1.90)	0.371
Vitamin B1	(mg)	0.93	±	0.24	0.69	±	0.12	0.76	(0.61 to 0.92)	0.91	±	0.19	0.67	±	0.10	0.69	(0.62 to 0.94)	0.873
Vitamin B2	(mg)	1.14	±	0.25	1.14	±	0.35	0.96	(0.79 to 1.25)	1.16	±	0.25	1.27	±	0.31	1.02	(0.91 to 1.28)	0.210
Folate	(µg)	346	±	103	334	±	110	1.02	(0.70 to 1.24)	342	±	83	390	±	122	1.14	(0.90 to 1.35)	0.126
Vitamin C	(mg)	136	±	62	109	±	45	0.85	(0.53 to 1.08)	138	±	54	126	±	42	0.94	(0.65 to 1.22)	0.247
SDF	(g)	3.1	±	0.8	2.0	±	0.6	0.63	(0.48 to 0.80)	2.9	±	0.5	2.0	±	0.5	0.70	(0.53 to 0.87)	0.510
IDF	(g)	10.9	±	2.9	8.0	±	2.1	0.74	(0.61 to 0.91)	10.5	±	1.6	7.9	±	2.0	0.76	(0.58 to 0.93)	1.000
TDF	(g)	14.9	±	3.8	10.9	±	3.0	0.68	(0.60 to 0.93)	14.3	±	2.0	10.4	±	2.5	0.71	(0.59 to 0.90)	0.939
Median								0.87	(0.77 to 0.99)							0.86	(0.80 to 0.99)	0.613

Abbreviations: DR, dietary record; FFQ, food frequency questionnaire; IDF, insoluble dietary fiber; MO n-3 PUFA, marine origin n-3 PUFA; MUFA, monounsaturated fatty acids; PUFA, polyunsaturated fatty acids; SD, standard deviation; SDF, soluble dietary fiber; SFA, saturated fatty acids; TDF, total dietary fiber. [a] Difference = (FFQ / 7-day DR). The ratios are shown median (interquartile range). [b] The Mann-Whitney U test was used to compare measurement error between participants aged <75 and ≥75 years. Bold values are statistically significant ($p < 0.05$). [c] Value are shown as energy-dense macronutrient (% energy intake). [d] Sum of eicosapentaenoic acid (20:5), docosapentaenoic acid (22:5), and docosahexaenoic acid (22:6). [e] Sum of retinol, β-carotene/12, α-carotene/24, and cryptoxanthin/24.

Table S3. The coefficients of variation, and within- to between-person variance ratios of energy and nutrient intake based on dietary record (DR), shown per sex [a].

	Women (n = 65)			Men (n = 78)		
	CV_w (%)[a]	CV_b (%)[b]	VR[c]	CV_w (%)[a]	CV_b (%)[b]	VR[c]
Energy	13.1	12.0	1.09	15.3	14.8	1.03
Protein	17.9	14.9	1.20	19.5	17.0	1.15
Fat	28.6	24.1	1.19	32.6	26.6	1.23
Carbohydrate	15.0	13.1	1.15	15.6	16.1	0.97
Protein [b]	15.5	10.6	1.45	14.5	10.8	1.35
Fat [b]	21.6	17.9	1.21	23.8	20.4	1.17
Carbohydrate [b]	10.5	8.1	1.30	12.0	10.6	1.13
SFA	35.2	29.7	1.19	37.2	31.8	1.17
MUFA	37.3	29.5	1.26	40.7	28.5	1.43
PUFA	32.5	23.2	1.40	37.2	30.1	1.24
n-6 PUFA	33.9	24.5	1.39	39.1	33.3	1.17
n-3 PUFA	48.1	32.8	1.47	48.6	30.0	1.62
MO n-3 PUFA [c]	105.7	65.6	1.61	93.7	57.7	1.62
Cholesterol	51.5	28.6	1.80	46.3	33.1	1.40
Iron	24.4	22.1	1.10	23.4	24.3	0.96
Calcium	30.8	27.8	1.11	30.6	34.9	0.88
Carotene	55.6	59.7	0.93	67.3	68.6	0.98
Vitamin A [d]	42.6	75.2	0.57	51.2	94.9	0.54
Vitamin D	94.6	52.3	1.81	84.9	54.6	1.56
α-tocopherol	29.7	25.4	1.17	33.1	29.5	1.12
Vitamin B1	29.1	23.5	1.24	31.4	23.2	1.35
Vitamin B2	24.7	20.2	1.22	22.7	25.7	0.88
Folate	25.3	26.6	0.95	29.4	34.4	0.85
Vitamin C	44.7	42.3	1.06	47.0	58.2	0.81
SDF	29.8	25.4	1.17	31.1	34.9	0.89
IDF	25.4	23.8	1.07	25.8	29.9	0.86
TDF	24.3	21.6	1.13	24.9	28.3	0.88

Abbreviations: CV_w, coefficient of within-person variation; CV_b, coefficient of between-person variation; IDF, insoluble dietary fiber; MO n-3 PUFA, marine origin n-3 PUFA; MUFA, monounsaturated fatty acids; PUFA, polyunsaturated fatty acids; SDF, soluble dietary fiber; SFA, saturated fatty acids; TDF, total dietary fiber; VR, ratio of within- to between-person variance. [a] The between-person variance (σ_b^2) and within-person variance (σ_w^2) for intake were calculated using one-way analysis of variance. [b] Value are shown as energy-dense macronutrient (% energy intake). [c] Sum of eicosapentaenoic acid (20:5), docosapentaenoic acid (22:5), and docosahexaenoic acid (22:6). [d] Sum of retinol, β-carotene/12, α-carotene/24, and cryptoxanthin/24.

Table S4. Group size required to ensure accuracy of energy and nutrients intake with 95% confidence interval (CI) within the specified % deviation (D_0) of a group's mean from the group's usual ("true") mean intake based on dietary record (DR), shown per sex [a].

	Women (n = 65)				Men (n = 78)			
D_0	2.5%	5.0%	10.0%	20.0%	2.5%	5.0%	10.0%	20.0%
Energy	193	48	12	3	278	69	17	4
Protein	332	83	21	5	413	103	26	6
Fat	860	215	54	13	1086	272	68	17
Carbohydrate	243	61	15	4	308	77	19	5
Protein [b]	217	54	14	3	201	50	13	3
Fat [b]	485	121	30	8	605	151	38	9
Carbohydrate [b]	108	27	7	2	158	40	10	2
SFA	1303	326	81	20	1471	368	92	23
MUFA	1391	348	87	22	1518	380	95	24
PUFA	980	245	61	15	1408	352	88	22
n-6 PUFA	1077	269	67	17	1624	406	102	25
n-3 PUFA	2087	522	130	33	2002	500	125	31
MO n-3 PUFA [c]	9511	2378	594	149	7437	1859	465	116
Cholesterol	2133	533	133	33	1990	498	124	31
Iron	666	166	42	10	699	175	44	11
Calcium	1058	265	66	17	1324	331	83	21
Carotene	4088	1022	255	64	5681	1420	355	89
Vitamin A [d]	4593	1148	287	72	7143	1786	446	112
Vitamin D	7183	1796	449	112	6266	1566	392	98
α-tocopherol	937	234	59	15	1211	303	76	19
Vitamin B1	858	215	54	13	934	234	58	15
Vitamin B2	626	157	39	10	720	180	45	11
Folate	827	207	52	13	1256	314	79	20
Vitamin C	2329	582	146	36	3440	860	215	54
SDF	942	236	59	15	1342	335	84	21
IDF	746	187	47	12	957	239	60	15
TDF	648	162	40	10	875	219	55	14

Abbreviations: IDF, insoluble dietary fiber; MO n-3 PUFA, marine origin n-3 PUFA; MUFA, monounsaturated fatty acids; PUFA, polyunsaturated fatty acids; SDF, soluble dietary fiber; SFA, saturated fatty acids; TDF, total dietary fiber. [a] Group size of DR assuming single observation for each individual = $1.96^2 \times [(CV_b^2 + CV_w^2)/D_0^2]$, where D_0 = the specified % deviation of group mean from group usual ("true") mean intake. All values are group size. [b] Value are shown as energy-dense macronutrient (% energy intake). [c] Sum of eicosapentaenoic acid (20:5), docosapentaenoic acid (22:5), and docosahexaenoic acid (22:6). [d] Sum of retinol, β-carotene/12, α-carotene/24, and cryptoxanthin/24.

Table S5. Number of days required to ensure precision of energy and nutrients intake within specified correlation coefficient (r) between observed and usual ("true") mean intake based on dietary record (DR), shown per sex [a].

	Women (n = 65)					Men (n = 78)				
	0.75	0.80	0.85	0.90	0.95	0.75	0.80	0.85	0.90	0.95
Energy	1	2	3	5	10	1	2	3	4	10
Protein	2	2	3	5	11	1	2	3	5	11
Fat	2	2	3	5	11	2	2	3	5	11
Carbohydrate	1	2	3	5	11	1	2	3	4	9
Protein [b]	2	3	4	6	13	2	2	4	6	12
Fat [b]	2	2	3	5	11	1	2	3	5	11
Carbohydrate [b]	2	2	3	6	12	1	2	3	5	10
SFA	2	2	3	5	11	2	2	3	5	11
MUFA	2	2	3	5	12	2	3	4	6	13
PUFA	2	2	4	6	13	2	2	3	5	11
n-6 PUFA	2	2	4	6	13	2	2	3	5	11
n-3 PUFA	2	3	4	6	14	2	3	4	7	15
MO n-3 PUFA [c]	2	3	4	7	15	2	3	4	7	15
Cholesterol	2	3	5	8	17	2	2	4	6	13
Iron	1	2	3	5	10	1	2	3	4	9
Calcium	1	2	3	5	10	1	2	2	4	8
Carotene	1	2	2	4	9	1	2	3	4	9
Vitamin A [d]	1	1	1	2	5	1	1	1	2	5
Vitamin D	2	3	5	8	17	2	3	4	7	14
α-tocopherol	2	2	3	5	11	1	2	3	5	10
Vitamin B1	2	2	3	5	11	2	2	4	6	13
Vitamin B2	2	2	3	5	11	1	2	2	4	8
Folate	1	2	2	4	9	1	2	2	4	8
Vitamin C	1	2	3	5	10	1	1	2	3	7
SDF	2	2	3	5	11	1	2	2	4	8
IDF	1	2	3	5	10	1	2	2	4	8
TDF	1	2	3	5	10	1	2	2	4	8

Abbreviations: IDF, insoluble dietary fiber; MO n-3 PUFA, marine origin n-3 PUFA; MUFA, monounsaturated fatty acids; PUFA, polyunsaturated fatty acids; SDF, soluble dietary fiber; SFA, saturated fatty acids; TDF, total dietary fiber. [a] Number of days of DR = $[r^2/(1 - r^2)] \times$ VR, where r = unobservable correlation coefficient between observed and usual ("true") mean intakes of individual and VR = within-person/between-person variance ratio (σ_w^2/σ_b^2). All values are number of days. [b] Value are shown as energy-dense macronutrient (% energy intake). [c] Sum of eicosapentaenoic acid (20:5), docosapentaenoic acid (22:5), and docosahexaenoic acid (22:6). [d] Sum of retinol, β-carotene/12, α-carotene/24, and cryptoxanthin/24.

Table S6. Number of days required to ensure accuracy of energy and nutrients intake with 95% confidence interval (CI) within the specified % deviation (D_1) of an individual's usual ("true") mean intake based on dietary record (DR), shown per sex [a].

	Women (*n* = 65)				Men (*n* = 78)			
D_1	5%	10%	20%	30%	5%	10%	20%	30%
Energy	26	7	2	1	36	9	2	1
Protein	49	12	3	1	59	15	4	2
Fat	126	31	8	3	163	41	10	5
Carbohydrate	34	9	2	1	37	9	2	1
Protein [b]	37	9	2	1	32	8	2	1
Fat [b]	72	18	4	2	87	22	5	2
Carbohydrate [b]	17	4	1	0	22	6	1	1
SFA	191	48	12	5	212	53	13	6
MUFA	214	53	13	6	255	64	16	7
PUFA	162	41	10	5	213	53	13	6
n-6 PUFA	177	44	11	5	235	59	15	7
n-3 PUFA	356	89	22	10	362	91	23	10
MO n-3 PUFA [c]	1717	429	107	48	1348	337	84	37
Cholesterol	407	102	25	11	329	82	21	9
Iron	91	23	6	3	84	21	5	2
Calcium	146	36	9	4	144	36	9	4
Carotene	475	119	30	13	697	174	44	19
Vitamin A [d]	279	70	17	8	402	101	25	11
Vitamin D	1375	344	86	38	1108	277	69	31
α-tocopherol	135	34	8	4	169	42	11	5
Vitamin B1	130	32	8	4	151	38	9	4
Vitamin B2	94	23	6	3	79	20	5	2
Folate	98	25	6	3	132	33	8	4
Vitamin C	307	77	19	9	339	85	21	9
SDF	136	34	9	4	148	37	9	4
IDF	100	25	6	3	102	26	6	3
TDF	91	23	6	3	96	24	6	3

Abbreviations: IDF, insoluble dietary fiber; MO n-3 PUFA, marine origin n-3 PUFA; MUFA, monounsaturated fatty acids; PUFA, polyunsaturated fatty acids; SDF, soluble dietary fiber; SFA, saturated fatty acids; TDF, total dietary fiber. [a] Number of days of DR = $(1.96 \times CV_w/D_1)^2$, where D_1 = the specified % deviation of individual mean from usual ("true") mean intake. All values are number of days. [b] Value are shown as energy-dense macronutrient (% energy intake). [c] Sum of eicosapentaenoic acid (20:5), docosapentaenoic acid (22:5), and docosahexaenoic acid (22:6). [d] Sum of retinol, β-carotene/12, α-carotene/24, and cryptoxanthin/24.
